# A Wicked Problem: Early Childhood Safety in the Dynamic, Interactive Environment of Home 

**DOI:** 10.3390/ijerph10051647

**Published:** 2013-04-24

**Authors:** Jean Simpson, Geoff Fougere, Rob McGee

**Affiliations:** 1 Injury Prevention Research Unit, Department of Preventive & Social Medicine, Dunedin School of Medicine, University of Otago, Dunedin 9054, New Zealand; 2 Department of Public Health, Wellington School of Medicine, University of Otago, Wellington 6021, New Zealand; E-Mail: geoff.fougere@otago.ac.nz; 3 Department of Preventive & Social Medicine, Dunedin School of Medicine, University of Otago, Dunedin 9054, New Zealand; E-Mail: rob.mcgee@otago.ac.nz

**Keywords:** childhood, injury, parents, risk perception, social environment, qualitative research

## Abstract

Young children being injured at home is a perennial problem. When parents of young children and family workers discussed what influenced parents’ perceptions and responses to child injury risk at home, both “upstream” and “downstream” causal factors were identified. Among the former, complex and interactive facets of society and contemporary living emerged as potentially critical features. The “wicked problems” model arose from the need to find resolutions for complex problems in multidimensional environments and it proved a useful analogy for child injury. Designing dynamic strategies to provide resolutions to childhood injury, may address our over-dependence on ‘tame solutions’ that only deal with physical cause-and-effect relationships and which cannot address the complex interactive contexts in which young children are often injured.

## 1. Introduction

Many young children are unintentionally injured in predictable and preventable events occurring at home, a place we think should be safe. Early childhood injury is a major and persistent international public health problem [1] and unintentional childhood injury at home is a significant contributor [2,3,4,5]. New Zealand (NZ) ranked a dismal 24th out of 24 rich OECD countries for rates of childhood injury death [6]. A retrospective analysis of injury data has shown that while 23% of NZ’s 0–14 year olds were aged 0–4 years, this young group contributed 52% of the injury deaths and 35% of the injury hospitalizations for 0–14 years old [2]. Fifty-four percent of injury deaths and 53% of injury hospitalizations for the 0–4 years old occurred at home (the majority of which were coded unintentional). In comparison, road traffic crashes contributed 29% of injury deaths and 4% of hospitalizations for this age group [2]. Lack of success in limiting childhood injury has continued, possibly due to the gap constantly noted between research and practice, but potentially also because child safety is a more complex problem than we have recognized and requires further analysis to obtain greater insight into how to design, develop and deliver strategies more effectively. 

An examination of the descriptions of the circumstances of injuries indicated that around 20% of NZ early childhood home deaths could, theoretically, be prevented using proven passive interventions. The other 80% of injuries lie outside the operating range of these interventions. The rationale for prevention strategies used for many of the 80% is based on descriptive epidemiology, risk analyses, experience and intuition. Strategies are formally enacted through regulation, facilitated through government-funded safety programs, and informally disseminated through the generations, peers and the media. Proving success can be difficult, but potentially unsafe events may be serious for the child and the precautionary principle is invoked: do no harm, but where the child could be seriously harmed, doing nothing is not an option [7]. 

Passive interventions that do not rely on ongoing human action often operate at the regulatory level. Single causes of injury or a narrow band of circumstances are addressed by product or environmental modification or engineering. Some are not always effective. Proper swimming pool fencing separates the child from the hazard [8], but drownings still occur related to factors other than the physical environment. Events do not occur in isolation and the chains of events leading to children being injured may contain common circumstances regardless of the cause of injury. For example, a lack of active supervision, crowded households, cultural norms, housing conditions and living in deprived neighborhoods have been associated with child injury [9,10,11]. 

When examining childhood injury, researchers and practitioners bring different perspectives. For parents, injury prevention is usually a personal, family and peer-based experience while researchers often examine the population experience. The traditional epidemiological paradigm offers one set of research tools, but by using an ecological approach, alternative lenses may identify previously unrecognized factors for further examination. Examining the chains of events leading to a child injury or to protection from injury is likely to contribute to more effective designing and delivery of initiatives. 

Katcher *et al.*’s list of home hazards includes a lack of supervision [4] and two aspects have been noted as being instrumental in reducing young children’s exposure to injury risk: supervisors’ perception of, and response to, the risk of child injury [12]. Longitudinal studies suggest there are “upstream” and “downstream” factors influencing risk perception and response [9,13]. Downstream factors relate to the individual and family social and physical environments, while upstream factors encompass broader societal factors, for example social expectations, income distribution, employment and changes in family forms. 

Rittel and Webber’s “wicked problems” model recognizes these multiple “messy” dimensions of interactive relationships between factors such as social environments, human interaction and politics [14,15]. The origins of the model lie in urban planning where increasingly complex issues were being faced by planners once simpler problems had been addressed. Contemporary expectations, philosophies, goals and values left a set of complex problems to define, set goals for and solve. The origin of the model parallels experiences in environmental health [15] and child safety. The wicked problem approach suggests that it is a mistake to try solving problems as engineers by focusing on a single cause and modifying its characteristics to remove the problem. Such “tame solutions” were not effective in resolving problems involving complex social factors and they considered it “morally objectionable” as well as impractical to not recognize “the inherent wickedness of social problems” (defined as “*wicked in a meaning akin to that of* “*malignant*” (*in contrast to* “*benign*”) *or* “*vicious*” (*like a circle*)*or* “*tricky*” (*like a leprechaun*)*or* “*aggressive*” (*like a lion*,*in contrast to the docility of a lamb*)” [14]). 

In this study, practitioners of child care (parents and those who work closely with young families) were asked—as legitimate and experienced observers and commentators—about the influences on perception of, and responses to, early childhood injury risk at home. This paper reports on a set of upstream themes that emerged from their narratives, and reflects on how these might affect the design and delivery of relevant strategies. While the modified Haddon’s matrix offers a cause-specific analysis that encourages identification of upstream factors [16] it does not operate over the wider spectrum of injury events occurring at home. We apply the “wicked problems” framework to help interpret the findings [14]. 

## 2. Methods

Two closely linked research questions were framed, namely: what influences parents’ perceptions of the risk of injury to a young child at home, and what influences parents’ ability and willingness to respond appropriately to minimize that risk? The methodology chosen was an interpretive constructivist approach [17] which offered a good fit for focusing on these questions without predetermining content boundaries. This facilitated examining expected, common experiences and meanings, and unexpected or diverse views, beliefs and experiences of people who are actively involved in safety management for young children. An underlying question was the definition of injury and who defines and describes the problem. To date, the definition has predominantly been that of the epidemiologists. Their use of the data is different to that of hospital management wanting to know the “burden of injury” to prioritize resource allocation, which is different to that of clinicians working with the individual child or those in community assessing emergent issues. For example, counting injuries that “nearly happened” is not useful for the epidemiologist, whereas these events provide crucial learning tools for parents and those working with parents. 

The study was conducted in Hawke’s Bay, New Zealand. Critical support was provided by people in the health sector and the advisory group (Te Roopu) established with local indigenous iwi (Māori tribe) and health providers. The region has a wide socioeconomic spread and a high Māori population (NZ/Aotearoa’s indigenous people).

Two sets of participants were recruited. One comprised of health care professionals, home visitors, nannies, social workers and community educators (collectively referred to as family workers) from each of the local service providers who worked with young children and specifically included injury prevention as part of their practice. The management for each provider was approached, the invitation extended and a time negotiated for holding a focus group discussion with available staff. The second set of participants comprised parents of children <5 years old who had sought medical treatment for a home injury. Recruitment was undertaken by staff at the public hospital emergency department and the two urban medical centers where families commonly sought treatment. During the waiting or treatment time, the parent was invited to participate and those willing to do so provided contact details for the interviewers. Ethical approval was granted by the Central Regional Health and Disability Ethics Committee.

Eighteen individual interviews and six focus groups were conducted face-to-face. Interviews, which took about an hour, and focus group discussions, which took approximately two hours, were audio-recorded and transcribed. Parent interviews were held within two weeks of the injury incident to minimize loss of recall [18]. Interviews with Māori parents were conducted by an experienced Māori health worker, and other interviews were conducted by the first author who also facilitated the focus group discussions. A previously-piloted qualitative semi-structured interview with open-ended questions was used with both sets of participants [18]. All but one parent chose to meet at the child’s home. Using responsive interviewing, a conversational exchange was created to foster a relationship so in depth information was obtained [19]. One parent interview was excluded from further analysis because it emerged during the interview that it was not a home injury.

The individual parent interviews began with the injury the child had sustained. Participants were encouraged to reflect on their own experience and that of peers and family, with prompts used when necessary to expand participants’ explanations or commentary. The focus groups, with between two and eight participants, drew more on their professional experience for their discussion. 

The narratives transcribed were independently coded deductively, with codes based on factors in the child injury prevention literature, and inductively, to create new themes, by the first author and a second researcher. The coding was agreed following negotiation of any differences. The themes identified were categorized into “upstream” and “downstream” influences, that is, those that were related to the individual and those that were related to the community or society more broadly [13]. The findings and themes were discussed with the health sector advisors, the service providers and Te Roopu. 

With the complexity of the material gathered, possible models were considered for the upstream factors and Rittel and Webber’s wicked problems model was chosen to frame the analysis [14]. Wicked problems have ten distinguishing features and these are discussed below. Briefly, wicked problems have no definitive formulation, no stopping rule and solutions are not true-or-false, but good-or-bad. They do not have an immediate or ultimate test for a solution, and their complexity means that solutions are a “one-shot operation” often without the opportunity to learn by trial-and-error. Each wicked problem is essentially unique but can be considered a symptom of another problem. The choice of explanation for discrepancies can be explained in numerous ways and will determine the nature of the resolution. Although not all apply equally well to child home safety, the features of model offered a “good fit” for the themes, which were also explored with reference to additional literature, often from outside injury prevention. 

## 3. Results

Responses from family workers and parents are incorporated in the following exploration of the influences on parental perception of, and response to, injury risk to young children. Views differed, so while the themes have considerable common ground, where there are differences between the two sets of participants, these are noted. The findings from the narratives are presented in the following themes. The first two, fatigue and learning to parent safely, are ostensibly downstream factors. However, because they are influenced by a range of factors such as neighborhoods, governmental infrastructure, changes in community, social norms and expectations, income, employment, and the value of children in society, they provide a valuable starting point for examining upstream themes. 

### 3.1. Downstream Influences

#### 3.1.1. Fatigue

Parental fatigue influenced the perception of risk and the response to it. Continually interrupted sleep or lack of sleep, sometimes due to sick children, and often compounded by work or time commitments, resulted in sleep-deprived parents unable to make up for lost sleep. As one mother noted:

“*There are times when I*’*ve put her on the floor and I lay down on the floor and then I*’*ve dozed off, ... [I*’*m] just so tired*”.

(mother in paid work)

Lack of sleep was considered likely to lead to poor judgment, including the anticipation of and response to the risk of injury. Experienced family workers suggested that chronic tiredness and stress could also lead to a mother becoming desensitized, and had serious implications for risk perception and response. Family workers also had concerns about fatigue in the context of abusive relationships or disadvantaged households:

“*Sleep deprivation: when you*’*ve lost an hour*’*s sleep here, you lose two hours*’*sleep tomorrow night, you lose an hour*’*s sleep the next night—you don*’*t catch up on Friday. If you keep doing that, and you*’*ve got to deal with an abusive partner, [and] you*’*ve got no money…*”.

(family worker)

Being tired may be “part and parcel” of being a mother, but a number of participants thought contemporary mothers experience greater levels of tiredness than previous generations. While the level of manual labor of previous generations was acknowledged, possible current factors include being older (on average), and the demands generated by external schedules associated with paid work, the changing nature of the family and societal expectations. 

Fatigue was linked to parents working more paid hours than they would like. This affected professionals trying to maintain a career, which can include continuing to prove yourself in the workplace while managing a young family. It equally affected those in low paid employment, commonly with inflexible work hours or uncertain security that meant taking time off threatened their continuing employment but not taking time off was a threat to keeping young children safe. 

#### 3.1.2. Learning to Parent Safely

“*Babies aren*’*t cats or dogs or whatever that you chuck outside and leave for a couple of hours and they*’*ll be OK*”.

(mother with two children)

Parenting is a complex task and participants joked about a paid job being much easier, especially as parenting came “*without a manual*”. A direct relationship was drawn between nurturing and understanding child development and perceiving and responding appropriately to children’s injury risk. Safety involved knowing the child and actively supervising them, including anticipating the unexpected: “*…you get in the mode of thinking about what could happen: even though sometimes it fails you*” (*A mum*)*.* The time parents spent with the child was considered to influence their anticipation of a child’s actions and the consequences. Less time with a child could mean that while parents perceived risks and removed them, they may be less experienced in managing risks likely to occur with their child’s stage of development or temperament. 

Family workers also saw repeated patterns of high risk when parents believed their child to be more capable than (s)he was and the child was expected to do as he was told, remember instructions, and comply with rules (a set of family “regulations” which many adults would find difficulty adhering to). 

The quality of parent mentoring similarly influences perception and response to injury risk. Parents frequently identified members of their family particularly mothers, as mentors, also referring to early childhood services. Those working with families in disadvantaged environments identified difficulties upstream with gaps in the infrastructure for providing sufficient professionally trained home-based services negatively affecting parents’ supervisory skills. Conversely, negative judgments on parenting skills can affect parents’ confidence to make good safety decisions. This was one reason why the overabundance of parenting books on sale providing overwhelming, and sometimes conflicting, advice on parenting was considered unhelpful by so many of the participants. A number of participants opined that literate parents-to-be were a captive market for publishers. 

Another conflict emerged between the public message, “injures are predictable and preventable” and medical professionals acceptance that unintentional injury does occur. Although apparently at odds, the pragmatic response of the latter was seen to enable parents, who knew the safety messages, to avoid being paralyzed by their failure to keep their child safe. 

### 3.2. Upstream Influences

#### 3.2.1. Changes in the Neighborhood

Almost all participants remarked on the changing face of the community over time and saw the changes as influencing perception and response to injury risk:

“*[In the past] there were more people in the community. Drive down the street now, there*’*s actually no one at home because everybody*’*s out working...so it*’*s quite different*”

(a suburban mother)

Childhood memories of ranging the neighborhood without parental constraint (and without harm) were recalled. Freedom and lack of harm may have been an illusion, as observant community members (the “nosy old lady”) may have provided covert supervision, influencing some activity. However, communal responsibility has changed and participants perceived effects such as fewer informal minders present in neighborhoods, suspicion of outsiders watching children, less community trust, and more reluctance to be involved in other peoples’ lives. A current phenomenon reported was families living in neighborhoods which were not “their” neighborhood because their social and recreational activities occurred elsewhere. Such disconnection was seen as potentially negative by some mothers because it could isolate the primary care-provider at home and did not provide a “yardstick” for normal child behavior or actual risk. The dominant discourse among participants was that protecting young children from serious harm was a community priority. An overt collective responsibility was a positive influence because it indicated that society took child safety seriously and that children being safe was the norm. 

#### 3.2.2. Expectations and Norms

Parental perception and response to risk is influenced by what constitutes an acceptable injury. It is unclear whether the threshold had changed over time, but family workers suggested that a reduction in family size and fewer children dying from communicable diseases, and increased assisted fertility, means that children may be seen as more precious (and costly), so that for some sectors of the population, greater care is exercised influencing current perceptions of and response to risk. Earlier generations may have been accepting of the long term consequences of injury, but this is not to say that less effort was expended in prevention. Many participants recalled that safety was a high priority for their parents and anything more serious than minor bruises and grazes was unacceptable. 

Negative influences were raised, one being an increased tendency to blame someone else when your child is injured. Not negating the collective responsibility, an alternative view was that “blaming” was an example of parents not being actively engaged to anticipate risk for their child and responding appropriately. 

Aspects of the changing norms over the last decade were thought to be negatively influential on parents’ perceptions of child development and safety. Increasing patterns of unsafe behavior associated with alcohol by teenagers and young women were a major concern to family workers. These seriously impair young mothers’ perceptions and responses to risks for their child. The continued marketing of pre-mixed fruity flavored alcoholic beverages and the lowering of the drinking age to 18 years were cited as having a very negative influence on child safety.

A further negative influence noted was a perceived reduction in personal involvement as a parent with the child. Some participants suggested a lack of time or opportunity to learn to be a parent, or a lack of confidence in taking on the role. Many participants were vocal about society’s negative expectations, seen through the media, reinforcing the “supermum” image. There was a sense that mothers especially were being judged and being ‘failed’ which was counter-productive to managing child safety.

“*The bar*’*s too high, you know. You gotta have perfect kids and perfect … manners and, all those things. It*’*s just constantly trying to strive to get that. And have the perfect family as well you know? The perfect car, to have them in the right school … It*’*s become such a competitive, stressful thing to have children*”.

(a mother with two children)

#### 3.2.3. Income and Expenditure

Two themes were identified in the narratives directly relating to income and expenditure. One was a recurring observation that our materialistic society was a negative influence on parents’ managing risk and creating a safe environment for their children while the second was the negative effects of insufficient income. 

Many participants feared that parenting was no longer a joy, but more of a “grind” than it had been in the past. This could be a symptom of the “me” generation, less willing to put the child first, having financial commitments, and a sense there are “*… high expectations now, people are too greedy, want the big house and both parents work. [It*’*s] not as simple as it used to be*” (*family worker*)*.* The “me” generation attitude, with the philosophy of self-first, was a particular characteristic of materialism that was seen as detrimental to child health and safety and spending time on the children would be in direct competition with personal time and purchasing power. Examples were given of parents at every income level, spending on themselves (clothes and alcohol) and on new “toys” (car, boat, television) but not on improving child safety. While cost might be a problem for some, it is used as an excuse for inaction. It was suggested that contemporary parents wanted to have the best of everything for themselves: jobs, income, and career. Earlier generations, particularly in the 1950s and 1960s were perceived as wanting to have, and do, the best for their children. Having no money did not prevent them from solving problems, and the ability to problem solve was seen as a positive influence for responding appropriately to risk.

Despite this ingenuity, it helps to have “*resources [so] that you can buy your way out of problems*” (*family worker*) and insufficient income is a negative influence on parents ability to control their environment, and therefore child safety. Parents and family workers raised the following concerns. Financial security is most unpredictable when a family is young and while home ownership may confer the right to make safety changes; many parents do not have discretionary money. For those in rental accommodation, the ability to control the environment can be much harder. Young families are often in rental accommodation, much of which is older stock. Some landlords are unwilling to make any improvement and some actively avoided making changes because that would require costly compliance with building regulations. Employment for some parents, especially in lower skilled jobs, does not pay sufficiently to support family life. The hours of work required to achieve a living wage, and the lack of flexibility in those hours are detrimental to family wellbeing and child safety. Although parents in professional positions are also under pressure, they were considered more likely to be able to negotiate or reorganize their time to be available for their children. 

#### 3.2.4. Work-Life Balance

Work-life balance was noted as an important influence on parental responses to child safety with positive and negative facets affecting their safety management. There are societal expectations that women will be in the paid workforce and an increasing norm that families need two incomes to make ends meet. Mothers’ mental well-being, and therefore the safety of the child, was seen as being closely influenced by work-life balance. A few parents had deliberately opted to spend more time with their children by reducing their paid work hours, or taking leave:

“*It*’*s mostly about whether you want the money or whether you want the lifestyle and your kids. And for us it*’*s much more important for us to be around and have the lifestyle with the kids really*”.

(parent of three children)

These participants were more likely to describe the option as a luxury, a “perk” of the job; sometimes with the rider of being fortunate to have the choice, despite peers sometimes finding their choice inexplicable, not least because of the level of hard work being a full time parent demands. For others, however, there was no easy option. One mother needed to be working in her profession for her mental health, but no position was available. Another’s partner worked away from home during weekdays leaving her sole parent. For a third, the family needed her income to survive, so it was immaterial that she wanted to be home. This situation was also thought common among lower paid women and was a problem for adequate child supervision and safety. In family workers’ experience, women in unskilled positions had limited control over their working hours, the jobs were often casual so did not cover sick leave, and older siblings were left babysitting unsupervised. 

#### 3.2.5. Value of Parents and Children

The strength of the participants’ observations that NZ society does not value its children was surprising. The lack of the following: parenting training, financial recompense for parenting, a living wage, acknowledgement of those who do a good job and adequate parental leave, conveyed the message that parenting was not important. Further undervaluation of children noted was the low pay for early childhood workers. Incentives in NZ policy that actively penalize mothers of young children who do not move into the paid workforce were also seen as potentially negative. Considerable value was seen in being part of the workforce, but requiring young mothers to take up unskilled work in preference to training and work that increased their parenting skills was seen to value the fiscal dollar over investing long term in parenting and greater child safety and another indication that parenting was not considered a legitimate job that contributed to the economy. 

### 3.3. Analysis of Child Safety as a Wicked Problem

The complexities identified in the participants’ narratives, and subsequent analyses, are reflected in many of the ten features of the wicked problem which, therefore, offered a framework for exploring themes arising from the influences on parental perception of and response to the risk of child injury at home. These are presented in Table 1, followed by a discussion of key emergent themes for early childhood safety in light of current knowledge of upstream factors. 

## 4. Discussion

The participants’ narratives around influences on perception of and response to injury risk linked factors that affect early childhood safety with problems requiring solutions at a societal level. The following section draws links between participants’ identification of the complex and interactive nature of these influences and factors identified in the literature. 

**Table 1:** Summary of differences between Rittel and Webber’s [14] wicked and tame problems with examples for child home safety.

	Features of a wicked problem	Tame problems (generic)	The wicked problem of child home safety
1	*There is no definitive formulation of a wicked problem*	*The definition of injury and specific causes are determined by experts using scientific data. Solutions are developed from these.*	The definition of an injury worthy of attention is not agreed. What stakeholders consider important varies and measures of the outcome of effort are not agreed.
2	*Wicked problems have no stopping rule*	*The task is done when the problem has been solved.*	Some aspects of child injury are tame and can be solved with tame solutions. The less easily solved are influenced by ongoing social change in families, society values, and economics, which affect the context in which parents are managing child safety.
3	*Solutions to wicked problems are not true-or-false, but good-or-bad*	*The problem can be either solved or not solved, with partial solutions able to be defined.*	How the problem is solved, and whether it is a good solution or not, can depend on cultural expectations, child-rearing patterns, and societal influences. What is considered an acceptable outcome differs, depending on the perspective, e.g., experts or parents.
4	*There is no immediate and no ultimate test of a solution to a wicked problem*	*Interventions can be developed and trialed to show their efficacy and piloted to test for effectiveness.*	Different definitions of injury worthy of attention are used by different stakeholders, so the outcomes sought may vary and so too will the solutions.
5	*Every solution is a* “*one-shot operation*”*: there is no opportunity to learn by trial-and-error, every attempt counts*	*Interventions can be trialed and piloted.*	Some solutions required are related to social change, and changes in social policy occur at the national level, and cannot be piloted or trialed with a small population first, e.g., changes in welfare benefits paid, changes in taxation, provision of health services.
6	*Wicked problems do not have an enumerable* (*or an exhaustively describable*)*set of potential solutions, nor is there a well-described set of permissible operations that may be incorporated into the plan*	*The problem can be mediated or modified by scientifically based processes. There are patterns of problem solving that are transferable.*	The interaction of various factors, child and adult behavior, social and physical environments, neighborhoods, family patterns, cultural differences and society expectations, means a complex context that can affect child safety directly, but also indirectly. The influence is not necessarily consistent, so predicting specific risk or protective factors can be difficult.
7	*Every wicked problem is essentially unique*	*The problem can be solved by addressing it as other problems have been, most commonly by addressing the mechanism that causes the energy force that result in injury.*	Child injury at home has unique features compared to other injury problems because young children are vulnerable and are dependent on adults for their environment. In addition, young children have not developed the cognitive function and/or the mobility skills to mediate or moderate their environment safely.
8	*Every wicked problem can be considered to be a symptom of another problem*	*The problem can be treated as a discrete event independent of others.*	Child home injury may reflect other existing problems: fatigue, stress, poverty, social deprivation, family dysfunction, lack of social support, lack of knowledge of parenting, child development, safety, risk or management.
9	*The existence of a discrepancy representing a wicked problem can be explained in numerous ways. The choice of explanation determines the nature of the problem*’*s resolution*	*The problem can be presented and regardless of the perspective, there is little disagreement.*	While experts define the problem, others who are responsible for keeping children safe have differing views about what is acceptable or otherwise; ranging between over protection and lack of experience of managing risk, and the view that injury is how children learn to be safe and this should not be mediated by adults except in extreme cases.
10	*The planner has no right to be wrong*	*The presentation of specific solutions to specific problems is proven by* “*scientific*” *evidence, and therefore considered to have been stringently tested. Other stakeholders are in accord.*	The complexity and interactive nature of the problem means that questions are unanswered by scientific processes. A lack of certainty in science, however, is not an excuse for doing nothing. Child injury can become an emotive issue in the public perception, either for too much or too little response at the individual or the population level.

Personal fatigue was a recurrent theme in this study, with participants noting as research has, that lack of sleep affects mental acuity, competence, aggression and the ability to make good judgment, all of which matter for child safety. Further complexity is introduced with fatigue as a symptom of depression [20], which has been linked to child injury [21]. Sleep disturbance among young children is associated with child injury [22,23] and contributes to parental sleep deprivation. In addition, New Zealanders work comparatively long hours [24] so parents may feel “time-poor” and for women, long working hours combined with the “second shift” (unpaid hours of mothering and housework) may result in high levels of ongoing fatigue [25]. Maternal welfare suffers as mothers generally protect time with children at a cost to themselves [26] and mothers are having less sleep than is recommended for maintaining mental and physical health [26].

The child development literature supports participants’ insistence that a caring, engaged and constant adult with whom the child can develop a relationship is important [27]. Measuring supervision is difficult [28,29], yet supervision is necessary in a caring relationship, and is beneficial to child safety [30,31,32]. Active supervision forms the “glue” of caring by a “conscientious parent” [33]. Tacit caring skills identified in other environments are attributes of such supervision: anticipating and defusing potential upsets, ensuring those unable to care for themselves are not left “hungry, angry, lonely or tired”, having consistent and fair boundaries, and keeping everyone safe emotionally and physically [34].

The participants’ observations on changes in family concurred with findings of a social gradient for child injury in NZ [35], concerns over older siblings’ home responsibilities and the lack of mentoring for young supervisors [36,37]. Compared to earlier generations, a greater proportion of NZ marriages now end in divorce, single-parent families are increasing [38] and the median maternal age for the first baby has risen from 26.8 years in 1986 to 30.7 in 2006 for nuptial births. Fewer children are being born, but more children are born to lower compared to higher income households [39]. Families in NZ are relatively mobile and high housing costs contribute as people opt for cheap, poor-quality, rental housing. In the face of poverty, other families combine households which can increase overcrowding, a risk for child injury [40]. 

Participant narratives reflected concern about increasing disparity between the overworked and underworked and its implication for child safety, an observation shared by Australian academics:

“*We have seen a growth in high-end jobs* (*mostly with very long hours*)*, in low-end jobs* (*mostly with not enough hours*)*and in no jobs at all for a sizeable group of men who don*’*t succeed in the formal education system. Very little of this is good news for children*”.

[41]

Post World War II, NZ was considered an egalitarian society with good child health, and families commonly living on a single wage supplemented by a universal child benefit. Now housing costs are high and household survival often requires more than one income. Some assistance is available for low-paid employed parents and there is direct provision of services and early childcare, however, benefits are means-tested and not pegged to inflation (unlike superannuation for older people). Parental leave in NZ is 14 weeks, much less generous than Sweden (68 weeks) or the UK (39 weeks). The International Social Survey Programme conclusion that inequality rose faster in NZ than in most of the Western world [42], with more emphasis placed on “*personal aspiration and responsibility, and less on collective well-being and support from the state*” [43] supports participants’ concerns about changing values and widening disparities. A large gap between rich and poor is evident in NZ [44] with a “*large shift in income from low and middle-income groups to the highest income group*” noted prior to 2000 [45]*.* Recent data show 20% of dependent children in NZ live in significant or severe hardship compared to 4% of those aged 65+ years [46] (these reports use international standardized categories for measuring relative poverty). Such inequalities impact on housing and child safety [47].

Traditional economic theory defined unpaid work, undertaken predominantly by women for their own families and households (providing food, maintaining the household, bringing up and keeping children safe, and caring for others) as “unproductive”, and the role of parents, especially for women, is devalued [33,48]. Although seriously criticized for the inequity it supports [49], it appears to continue as an underlying assumption behind policy decisions, despite the estimated value of this unpaid work to NZ being of the order of 39% of gross domestic product [50]. 

Work-life balance has changed in recent decades [51] and Hakim’s suggestion that most women prefer an adaptive lifestyle, combining work and home [52] fitted many of the participants’ narratives. Women have increased control over fertility, paid work may be more flexible and employment is increasing, for example, in service industries where women are more likely to be employed [52]. However, the employment environment makes it harder for workers, both fathers and mothers, to be good parents [26,41,48]. Reduced job security and conditions do not support family life, and include casual contracts, longer hours and expectations that paid work will always come before family. NZ lacks recognition of young children’s needs for health and safety: “*Returning to work sooner than desired after having a baby, and working full-time rather than part-time, are consistent with women accommodating a job rather than the workplace accommodating mothers*” [53]. “*Institutional and structural factors*” such as a reduction in the hours required to work, and recognition that career paths may be different for women than for men would be helpful [53]. 

### Where to from Here? 

Injury prevention moved away from “blaming the victim” prior to the 1970s, recognizing that victims were often unable to modify the unsafe features of their physical environment. The focus was on developing “tame solutions” which have generated successes. Human behavior still contributes to injury occurring and its prevention, and the value of employing behavioural sciences has been increasingly recognized [54]. Such approaches have been particularly useful in investigating the parent/child relationship associated with child injury [55]. What is in little evidence is the investigation of societal and systemic factors in the context of injury. One of the few is Hanson *et al.*’s [56] injury iceberg, which contributes to the recognition of social context [57]. 

Exploring the influences on child safety through the eyes of practitioners identified societal values and systemic issues. Typical of wicked problems, they do not operate independently, but do overlap with other dimensions of children’s health. There would be considerable benefit in developing and utilizing collaborative strength to engage national decision makers on child safety.

Strengths of this study included the insights two critical sets of child safety informants provided and their accord with conclusions in other literature. Society influences how parents parent, and necessary change does not occur in isolation. Making the environment supportive for those caring for young children will be crucial. This study recognises the legitimacy of practitioners’ voices and their ability to identify the complexities of influences that influence the exposure to risk of early childhood injury. Methodologically, the semi-structured schedule ensured the focus remained on child safety, while the open-ended, responsive data collection method meant informants were not restricted by accepted knowledge. There were limitations. Participation was voluntary which may have reduced the variety of voices. Few of the mothers were in full time employment and few were single parenting. Family workers’ observations provided insights into their clients’ lives, but these were not their clients’ views. Participants’ narratives did not have the benefit of a “kaupapa Māori” analysis so an important world view was not included*.*

While further thought is required to determine how to address these broader issues, this investigation must not be an excuse for doing nothing. Trialling a specific intervention to change how society perceives and values children will not be possible because, as a wicked problem “*every solution is a* “*one-shot*” *operation there is no opportunity to learn by trial-and-error, every attempt counts*”*.* Complex action will be required to shift the focus in public policy and economic thinking to a position that actively seeks to foster child safety and wellbeing in all areas of government. 

Positive actions would be to:

(a) Undertake child impact assessments for all proposed NZ legislation and policy development, and retrospectively for existing legislation and policy, specifically addressing child safety.
(b) Reorient underlying perceptions from children as a social cost, to children as an asset for society’s future.
(c) Develop policy to increase job security for primary carers and institute a living wage to contribute to improving child safety by addressing low-paid employment, poverty and potentially the social gradient of child injury.
(d) Adequately resource parenting programs proven to be effective cross-culturally to be accessible to parents and families. Additional resources may be needed to develop, deliver and evaluate effective programs to meet the needs of families for whom current systems do not work.
(e) Develop programs to train community health professionals to mentor and support parents and their extended families so they are competent care-providers.

## 5. Conclusions

New Zealand once prided itself on being an international leader in child health, but now lags behind in providing a safe place for children to live. Examining the problem through the eyes of those whose work involves keeping young children safe on a daily basis, has identified dimensions of the problem that have been missed and reprioritised aspects that have been accepted as norms. Reframing child safety as a wicked problem recognizes the potential relationships with other problems, as well as upstream factors including employment and income distribution, the changing nature of neighborhoods and families, work-life balance and the value of children in society. This is not to say that solutions such as swimming pool fencing or child resistant closures have not reduced child injury. However, unless the upstream influences on parents managing child safety are addressed, parents will continue to be victims of societal and systemic factors over which they have no control. Injury prevention has made considerable efforts to address the physical environment, but it is unlikely that resolutions will be achieved without challenging the societal factors that have been misdiagnosed, ignored or put in the “too hard basket”. 
